# Roles of Knowledge and Attitude in the Willingness of Nursing Students to Care for Older Adults in Hong Kong

**DOI:** 10.3390/ijerph18157757

**Published:** 2021-07-22

**Authors:** Winnie Lai-Sheung Cheng

**Affiliations:** School of Nursing, Tung Wah College, Hong Kong, China; winniecheng@twc.edu.hk; Tel.: +852-3190-6752

**Keywords:** attitudes, knowledge, willingness, nursing students, older-adult care, nursing education

## Abstract

Due to the ageing population, nursing students will be more likely to work with older adults after graduation. It is important to assess whether Hong Kong nursing students are well prepared to care for older adults. A convenience sample of 139 nursing students was surveyed using questionnaires: Palmore’s Facts on Ageing Quiz (FAQ), Kogan’s Attitudes Toward Old People scale (KAOP), and the Willingness to Care for Older People (WCOP) scale to assess the knowledge of and attitudes toward older adult care, and willingness to care for older adults, respectively. The overall score in the FAQ was medium-low (mean = 15.1, SD = 2.8). The KAOP score was medium-high (mean = 121.6, SD = 12.0). The willingness score was slightly high (mean = 5.2, SD = 1.1). Positive attitudes and knowledge about ageing are the predictors of nursing students’ willingness to take care of older adults. The findings provide evidence to nurse educators and clinical mentors that (a) courses providing knowledge about ageing are valuable, and (b) elements that cultivate positive attitudes towards older adult care should be included in curricula. Nursing curricula that provide knowledge and experience about older adult care play a pivotal role in creating a workforce of nurses ready and willing to care for the ever growing number of ageing adults.

## 1. Introduction

The world’s population is ageing [1]. Current demographic studies predict that, in 2030, approximately 28% of western Europe’s population, 21% of the US population [2], and 33% of the Hong Kong population will be 65 years or older [3]. Given these numbers, it is expected that a higher number of older people will use health and social services. After graduation, today’s nursing students, as the front-line workforce in health care service, are certain to be taking care of an increasing number of older adults at the primary and tertiary care levels, including residential and hospital care settings; this situation will be particularly true in Hong Kong (SAR of China). It is necessary to study the willingness of nursing students to work with older adults in Hong Kong. Yet, care of the elderly is the least preferred choice, with many studies reporting that nursing students have shown negative attitudes toward working with older adults [4,5,6].

Knowledge is fundamental to providing competent care. In Hong Kong, a minimum of 30 and 60 h, in theoretical and clinical practice respectively, in geriatric care is mandated to be included in undergraduate nursing curricula by the Nursing Council of Hong Kong [7]. Nursing students gain knowledge about common disorders and nursing care of older people from the nursing program. It has been reported that knowledge is positively related to working preference toward care of the elderly [8]. However, it is known that the willingness to take care of older adults is also influenced by attitude [8,9,10]. Attitude is a feeling about something or someone [11]. An individual’s intention (willingness) to perform an action is dependent on his/her attitude toward a given behavior [12]. Fishbein and Ajzen [11] further proposed that change in attitude affects behavioral intention. If nursing students embrace positive attitudes toward older adult care, they will be more likely to take care of older adults. In the previous two decades, numerous studies have been conducted to investigate nursing students’ knowledge of, and attitudes towards, older adults [4,5,6,8,9,13,14,15,16,17]. Nursing students generally displayed negative attitudes and were less motivated to undertake care of the elderly [6,8,9,17,18,19,20,21,22]. In China, Zhang et. al. [9] reported that positive attitudes toward older adult care and good knowledge about older adults among nursing students were found to be contributing factors to an increased willingness to work with older adults after graduation. Hong Kong, although a special administrative region of mainland China, has a different political system than China and a different culture, based on its history as a British colony. The attitudes of Hong Kong nursing students toward older adults may differ from those of nursing students in China. Different teaching styles in these two systems may be another factor affecting attitudes toward older adult care [23].

Little is known about Hong Kong nursing students’ knowledge of, and attitudes toward, older adult care. To improve the quality of care for older adults, it is important to assess the factors influencing the willingness of Hong Kong nursing students to care for older adults. In this study, both knowledge and attitudes of nursing students in this context were explored. This study also aimed to explore the factors predicting the willingness to work with older adults among nursing students in Hong Kong.

## 2. Methods

### 2.1. Design and Sample

This study was a cross-sectional descriptive survey. A convenience sample of nursing students from four nursing schools in Hong Kong was recruited. A total of 123 participants were required to test the association between variables, with 80% power, an effect size of 0.250, and a significance level of 5% [24].

### 2.2. Data Collection

The survey was carried out at the author’s institution. The data were collected from January to February 2018. Information was delivered via posters and Facebook to invite nursing students who were interested in joining the study. Informed consent was obtained after explanation of the study’s purpose and process, and data were collected face-to-face using self-reported questionnaires.

### 2.3. Variables and Instruments

The dependent variable (DV) was the level of willingness of nursing students in Hong Kong to take care of older adults. Inspired by a study on the willingness of medical students to choose a career in elderly care medicine [25], the level of willingness was measured using a 7-point Likert single-item scale, the Willingness to Care for Older People (WCOP), which is a subscale of the scale used by Zhang’s [9] study on nursing students’ care willingness towards the elderly. The answers could range from 7 for “strongly agree” to 1 for “strongly disagree”, thus, higher scores indicate greater willingness to undertake older adult care. Concurrent validity of the WCOP was demonstrated by the relationship with the positive attitude score (r = 0.735, *p* < 0.001), indicating that the dimension was consistent with expectations [10].

Independent variables (IVs) were knowledge about ageing, attitudes toward older adult care, and demographic characteristics. Knowledge about ageing was measured using a validated instrument, namely, Palmore’s Facts on Ageing Quiz (FAQ), which is a 25-item scale [26]. These 25 statements cover physical, mental, and social aspects of older people. The total possible score for the FAQ is 25. A higher total score indicates better knowledge of ageing. The factor loading ranged from 0.146 to 0.889 (CFI = 0.938) [27]. The internal consistency reliability was 0.850 [28].

Attitudes towards older adult care were measured using Kogan’s Attitudes toward Old People scale (KAOP) [29]. The KAOP comprises 34 statements which represent two subscales, namely Appreciation (KAOP+) and Prejudice (KAOP−), with Cronbach’s α of 0.81 and 0.83, respectively [30]. The factor loadings ranged from 0.410 to 0.880, and the two factors explained 54.7% of the variance [30]. The Appreciation subscale includes 17 positive statements that reflect favorable sentiments toward older people, such as “the elderly should have more power in society” and “the elderly are clean and neat”. The Prejudice subscale contains 17 negative items that reflect unfavorable sentiments, for example, “the elderly are unable to change” and “the elderly are irritable, grouchy and unpleasant” [29]. It is a 6-point Likert scale with a total summed score ranging from 34 to 204. A score of 136 shows a neutral attitude, the higher the score the more positive the attitude towards older adults. Cronbach’s α was 0.820 for the total scale [30]. Socio-demographic variables including age, year of study, education level, and clinical and personal experience related to older adult care were also assessed.

### 2.4. Statistical Analysis

All data were analyzed by the Statistical Package for Social Sciences (IBM SPSS) version 23.0. Differences in mean knowledge, attitude, and willingness scores between groups were tested with independent t-test and one-way ANOVA with Bonferroni’s post hoc test. Associations between variables were conducted using Pearson correlation. Multiple regression was performed stepwise on the dependent variable (i.e., nursing students’ willingness to work with older adults). Variables that were statistically significantly related to the nursing students’ willingness to work with older adults (i.e., knowledge about older adults, attitudes, and year of study) were entered into the model. In all statistical analyses, a *p*-value of less than 0.05 was considered as statistically significant. Prior analysis, missing values, normality, and outliers were checked, no outliers or missing values were found.

## 3. Results

A total of 139 participants completed questionnaires. Of the total number of participants, 75.5% were women and 24.5% were men. Demographic characteristics of the nursing students and the mean scores for knowledge about older adults, attitudes toward older adult care, and willingness to care for older adults by groups are presented in Table 1. There were no significant differences in mean knowledge, attitude, and willingness scores when participants were grouped according to gender, age, living history with older adults, and the frequency of volunteer activities for older adults; whereas, significant differences in some variables were found in groups according to years of study, attendance at gerontology nursing courses or gerontology clinical studies, and volunteer service for older adults.

Significant differences were found in mean knowledge and willingness scores among nursing students in different study years. Post hoc analysis using Bonferroni test revealed that year 5 nursing students had better knowledge scores than year 1 and year 2 nursing students. For willingness, the year 2 nursing students were less willing to take care of older adults than those studying in years 1, 3, and 5. Significant differences were reported in mean knowledge and willingness scores among nursing students according to whether they had attended gerontology nursing courses. Nursing students who had attended gerontology clinical study reported a better mean knowledge score. Nursing students who had joined volunteer activities for older adults also reported better knowledge and more willingness to work with older adults.

The overall mean knowledge, attitudes, and willingness scores are presented in Table 2. Overall, nursing students had a fair knowledge score. The attitudes toward older adults (KAOP) score was medium-high (mean = 121.6, SD = 12.0). The appreciation score (KAOP+) (mean = 64.4, SD = 8.1) was higher than the prejudice score (KAOP−) (mean = 57.2, SD = 8.2). The students had a fairly high score in willingness to work with older adults. Internal consistency of FAQ and KAOP was acceptable.

Table 3 shows the associations of demographic characteristics with knowledge, attitudes, and willingness. Significant relationships were found between age group and attitude, year of study and willingness, attendance in a gerontology nursing course and knowledge, attendance in a gerontology clinical study and knowledge, and volunteering activities for old adults and attitude. Table 4 reports the correlations between knowledge, attitude, and willingness. Significant relationships were found between knowledge and willingness, and attitude and willingness.

From the results shown in Table 3 and Table 4, knowledge, attitudes and year of study were significantly related to willingness. Multiple regression analyses were performed to further test if these variables are the factors predicting nursing students’ willingness to care for older adults. The results of the regression model indicate that the predictors explained 26.8% of the variance (R^2^ = 0.268, F(3, 135) = 16.503, *p* = 0.000), which is a good fit for the variables (Table 5). Stepwise regression reveals that attitude is the best predictor of the model followed by knowledge. Backward regression reveals that the model produced only a small difference in R square (R^2^ = 0.267) when year of study was removed. As we can see in Table 5, knowledge about ageing and attitudes had significant positive regression weights, indicating that nursing students with higher scores for these variables could be expected to be more willing to work with older adults. Year of study did not contribute to the regression model.

## 4. Discussion

This study attempted to explore the factors affecting nursing students’ willingness to care for older adults. The results of this study reveal that nursing students in Hong Kong are willing to work with older adults and that their attitudes towards older adults are positive. These findings are consistent with previous studies [9,31]. The findings of this study suggest that better knowledge about older adults and positive attitudes toward older adult care are associated with willingness to care for older adults among nursing students. The nursing students’ scores on the appreciation subscale were higher than on the prejudice subscale, which may explain why they are willing to work with older adults. This study also revealed that knowledge and attitude are the factors affecting their willingness to work with older adults.

According to the results of the present study, nursing students in year 5 have significantly better knowledge of older adults and are more willing to work with them. The explanation for this could be that a gerontology nursing course from which they gained theoretical knowledge about ageing was offered in the senior year of study. Furthermore, year 5 nursing students had completed a gerontology clinical study and the actual experience of working with older people may have influenced their willingness to care for them [32].

Clinical placement learning is an application of experiential learning. Through experiential learning, nursing students may have a chance to reflect on preconceptions of ageing via the incorporation of their concrete experience [33]. Exposing nursing students to the health issues and problems of older people provides them with an avenue for implementing nursing care to improve the quality of life for older people. This practical experience provides them the opportunity to transform their learning into an actualized professional role in a caring context. This process of experiential learning may help reconstruct their preconception of older people [34,35] and thus result in attitude change.

Clinical learning can be a good opportunity to inspire nursing students’ empathetic understanding of age-related changes and nurture their positive attitudes toward older adult care [34]. The findings of this study also indicate that nursing students who participated in volunteer activities with older adults had better knowledge of, and more willingness to care for, older adults. In this study, year 2 nursing students were less willing to take care of older adults than those studying in years 1, 3, and 5. When further analyses were performed, this group of nursing students reported that they did not attend a gerontology clinical study (n = 20), and nearly all of them (n = 19) reported that they had not undertaken a gerontology nursing course. These results support the idea that knowledge and clinical experience are key elements driving intention to work. Preparation of an adequate nursing workforce in older adult care is a cornerstone of providing quality care; nurse educators and clinical mentors of nursing students are in a good position to impart knowledge about older adults to nursing students and to develop related activities to increase their preference to work with older adults.

The present study reports that, for Hong Kong nursing students, knowledge about ageing is related to willingness to care for older adults, while it is unrelated to attitudes toward older adults. This finding is inconsistent with previous studies [8,36]. Further study is needed to conclude a causal relationship. In this study, the findings suggested that knowledge about ageing and attitude toward older adults are the predictors determining willingness of nursing students to take care of older adults in Hong Kong. This study also reports that attitude is the best predictor to determine willingness in nursing students to take care of older adults. The positive attitude towards older adults may be a mediating factor between knowledge and willingness to work with older adults [37]. This finding provides evidence to nurse educators and clinical mentors that key elements that could cultivate positive attitude towards older adult care should be included in curriculum designed to enhance nursing students’ willingness to work with older adults. To cultivate positive attitudes toward aged care among nursing students, concepts about care of older people should be embedded in the nursing curriculum earlier in their training.

Like other studies, there are limitations that need to be considered. First, the sample size was relatively small; a larger study involving more nursing institutions would confirm the results. Second, this cross-sectional design might not report the relationship among nursing students in different years of study; further study with a longitudinal design is suggested. Third, this study used a single question to measure nursing students’ willingness to work with older adults; a multi-item scale is suggested to elicit a deeper understanding of the reasons for working intention.

## 5. Conclusions

The results of this study demonstrate that, with better knowledge and positive attitudes, nursing students are more willing to work with older people. The nursing curriculum can supply information, while nursing educators and clinical mentors can instill positive attitudes by demonstrating empathy and care. Thus, nursing educators and clinical mentors may play a pivotal role in creating a workforce of nurses ready and willing to care for the ever growing number of older adults.

## Figures and Tables

**Table 1:** Demographic information and mean scores for knowledge, attitudes, and care willingness (N = 139).

				FAQ	KAOP	WCOP
		Number (%)		Mean (SD)		
Sex	Male	34 (24.5)		15.43 (3.10)	139.94 (15.34)	5.18 (0.99)
	Female	105 (75.5)		14.92 (2.67)	145.16 (16.51)	5.19 (1.15)
			*p*-value ^†^	0.361	0.105	0.949
Age	≤18	11 (8.0)		14.55 (2.69)	141.36 (22.2)	5.45 (0.93)
	19–23	115(82.7)		15.15 (2.69)	143.82 (15.47)	5.10 (1.13)
	24–28	12 (8.6)		14.67 (3.49)	145.83(19.82)	5.83 (0.94)
	≥29	1 (0.7)		13.00	156.00	4.00
			*p*-value ^‡^	0.736	0.808	0.090
Year of study	Year 1	28 (20.1)		13.75 (2.25)	142.68 (17.71)	5.39 (0.83)
	Year 2	20 (14.4)		14.13 (2.95)	142.60 (14.89)	4.30 (1.16)
	Year 3	28 (20.1)		14.70 (2.75)	144.61 (12.96)	5.32 (0.98)
	Year 4	19 (13.7)		15.11 (2.51)	149.32 (18.89)	5.16 (1.26)
	Year 5	44 (31.7)		16.49 (2.58)	142.43 (18.88)	5.16 (1.26)
			*p*-value ^‡^	<0.001	0.601	0.003
Gerontology nursing course	Yes	75 (54.0)		15.87 (2.68)	13.28 (1.53)	5.36 (0.94)
	No	64 (46.0)		14.06 (2.60)	19.40 (2.43)	4.98 (1.27)
			*p*-value ^†^	<0.001	0.668	0.047
Gerontology clinical study	Yes	64 (46.0)		15.74 (2.83)	142.66 (15.66)	5.31 (1.07)
	No	75 (54.0)		14.46 (2.61)	144.93 (16.92)	5.08 (1.15)
			*p*-value ^†^	0.007	0.415	0.221
Living with older adult	Yes	24 (17.3)		14.48 (3.20)	140.63 (11.90)	5.21 (1.14)
	No	115 (82.7)		15.17 (2.68)	144.57 (17.07)	5.18 (1.11)
			*p*-value ^†^	0.273	0.284	0.918
Lived with older adult in the past	Yes	52 (49.1)		15.61 (2.72)	146.31 (14.90)	5.31 (1.16)
	No	63 (50.9)		14.80 (2.61)	143.13 (18.68)	5.08 (1.07)
			*p*-value ^†^	0.110	0.322	0.275
Joined volunteer service for older people	Yes	110 (79.1)		15.35 (2.79)	145.11 (14.84)	5.34 (1.04)
	No	29 (20.9)		13.88 (2.46)	139.24 (20.70)	4.62 (1.21)
			*p*-value ^†^	0.01	0.085	0.002
Frequency of volunteer activities for older people	≥1 time per month	4 (2.9)		17.25 (2.96)	148.50 (8.70)	6.00 (0.82)
	2–4 time per month	6 (4.3)		15.08 (40.8)	156.00 (8.99)	5.33 (1.75)
	1 time per 6 months	29(20.9)		15.67 (2.85)	148.38 (14.47)	5.55 (0.83)
	≤1 time per 12 months	71 (51.1)		15.14 (2.65)	142.66 (15.13)	5.21 (1.05)
			*p*-value ^‡^	0.445	0.077	0.277

Note. ^†^ = independent t test; ^‡^ = one-way ANOVA; WCOP = Willingness to Care for Older People scale; FAQ = Palmore’s Facts on Ageing Quiz; KAOP = Kogan’s Attitudes toward Old People scale.

**Table 2:** Mean and SD of knowledge, attitudes, and willingness (N = 139).

	Mean	SD	Cronbach’s Alpha
FAQ	15.1	2.8	0.700
KAOP	121.6	12.0	0.769
KAOP+	64.4	8.1	
KAOP−	57.2	8.2	
WCOP	5.2	1.1	

Note. WCOP = Willingness to Care for Older People scale; FAQ = Palmore’s Facts on Ageing Quiz; KAOP = Kogan’s Attitudes toward Old People scale; KAOP+ = Appreciation; KAOP− = Prejudice.

**Table 3:** Correlation between demographic characteristics and knowledge, attitudes and care willingness (N = 139).

		FAQ	KAOP	WECS
		Pearson Chi-square (*p*-value)		
Sex	Male	0.064	0.544	0.689
	Female			
Age	≤18	0.548	0.016	0.389
	19–23			
	24–28			
	≥29			
Year of study	Year 1	0.334	0.347	0.012
	Year 2			
	Year 3			
	Year 4			
	Year 5			
Gerontology nursing course	Yes	0.017	0.575	0.172
	No			
Gerontology clinical study	Yes	0.026	0.456	0.261
	No			
Living with older adult	Yes	0.195	0.373	0.907
	No			
Lived with older adult in the past	Yes	0.780	0.656	0.256
	No			
Joined volunteer service for older people	Yes	0.103	0.015	0.082
	No			
Frequency of volunteer activities for older people	≥1 time per month	0.056	0.679	0.704
	2–4 time per month			
	1 time per 6 months			
	≤1 time per 12 months			

Note. WCOP = Willingness to Care for Older People scale; FAQ = Palmore’s Facts on Ageing Quiz; KAOP = Kogan’s Attitudes toward Old People scale.

**Table 4:** Correlation coefficient between knowledge, attitude, and care willingness (N = 139).

	FAQ	KAOP	WCOP
FAQ	1	0.044	0.188 *
KAOP	0.044	1	0.463 **
WCOP	0.188 *	0.463 **	1

Note. * *p* < 0.05; ** *p* < 0.001; WCOP = Willingness to Care for Older People scale; FAQ = Palmore’s Facts on Ageing Quiz; KAOP = Kogan’s Attitudes toward Old People scale.

**Table 5:** Correlations and results from the regression analysis (N = 139).

		Multiple Regression Weights		
Variable	Correlation with Willingness to care (WCOP)	b	β	F (3, 135)
FAQ	0.188 *	0.067 *	0.166	16.503 ***
KAOP	0.489 ***	0.023 *	0.252	
Year of study	36.832 *	0.024	0.033	

Note. * *p* < 0.05. ** *p* < 0.01. *** *p* < 0.001. WCOP = Willingness to Care for Older People scale; FAQ = Palmore’s Facts on Ageing Quiz; KAOP = Kogan’s Attitudes toward Old People scale.

## Data Availability

The data presented in this study are available on request from the corresponding author.
